# Visualization of Sampling and Ionization Processes in Scanning Probe Electrospray Ionization Mass Spectrometry

**DOI:** 10.5702/massspectrometry.S0078

**Published:** 2019-03-07

**Authors:** Bui Kamihoriuchi, Yoichi Otsuka, Aya Takeuchi, Futoshi Iwata, Takuya Matsumoto

**Affiliations:** 1 Department of Chemistry, Graduate School of Science, Osaka University, 1–1 Machikaneyama-cho, Toyonaka, Osaka 560–0043, Japan; 2 Faculty of Engineering, Shizuoka University, 3–5–1 Johoku, Naka-ku, Hamamatsu 432–8561, Japan

**Keywords:** ambient sampling and ionization, nano-volume liquid, liquid bridge, electrospray ionization, capillary probe

## Abstract

Ambient sampling and ionization techniques based on direct liquid extraction and electrospray ionization are of great value for rapid analysis and mass spectrometry imaging. Scanning probe electrospray ionization (SPESI) enables the sampling and ionization of analyte molecules in a solid material using a liquid bridge and electrospray, respectively, from a single capillary probe. To further improve SPESI, it is essential to understand the dynamic behavior of nanoliter volumes of liquids during sampling and ionization. In this study, the dynamic formation and breakage of the liquid bridge and the subsequent electrospray ionization were investigated by measuring the displacement of the capillary probe using a new optical technique. Measurements revealed that both the time from the formation of the liquid bridge to its breakage and the time from the breakage of the liquid bridge to the detection of analyte ions were correlated with the physical properties of the solvent. It was also found that both of these times were positively correlated with the flow rate. These results will not only lead to the improvement of sampling and ionization efficiencies but also afford a greater understanding of the physicochemical properties of charged nanoliter volumes of liquids.

## INTRODUCTION

Ambient sampling and ionization (ASI) techniques permit the rapid extraction and ionization of analyte molecules under atmospheric pressure and have advantages for direct analysis and mass spectrometry imaging (MSI). ASI techniques combining liquid-phase extraction and electrospray ionization (ESI)^1)^ have been reported since the early 2000s.^2)^ ESI utilizes the unique phenomenon in which molecules in a charged solvent are converted into gas-phase ions. By applying a high voltage to an analyte solution, charged droplets containing the analyte molecules are sprayed in the direction of the electric field between the apex of a Taylor cone and the mass spectrometry (MS) inlet. During the evaporation of these droplets, the repulsive Coulomb forces of charges eventually exceed the surface tension of the droplets, resulting in the generation of gas-phase ions. Since ESI allows large molecules to be ionized without fragmentation, it has been widely applied to biological samples. The representative ASI techniques are liquid microjunction surface sampling probe (LMJ/SSP),^3)^ desorption electrospray ionization (DESI),^4)^ liquid extraction surface analysis (LESA),^5)^ nanospray desorption electrospray ionization (Nano-DESI).^6)^

In 2012, we reported the technique of tapping-mode scanning probe electrospray ionization (t-SPESI).^7–9)^ In this method, a continuous flow of charged solvent is passed through a single vibrating capillary probe, and the probe position is adjusted to intermittently tap the sample surface. When the apex of the probe is close to the sample surface, a liquid bridge is formed between the two owing to the continuous flow of solvent from the end of the probe. Analyte molecules are extracted from the sample by the liquid bridge, and the resulting solution is transferred to the MS inlet by displacement of the probe and subjected to ESI from the end of the probe. In t-SPESI, the sampling and ionization processes are spatiotemporally separated owing to the probe vibration. Following the first report of SPESI, the influence of the probe geometry on the sampling and ionization was reported.^10)^ A similar method, sheath-flow probe electrospray ionization (SF-PESI),^11)^ was also reported in 2013.

We applied t-SPESI to MSI to visualize molecules on a sample surface. MSI of biological tissue sections permitted the ionization of various cancer-related molecules ranging from low to high molecular weight.^12)^ This was ascribed to the inhibition of ion suppression upon reducing the volume of liquid used for sampling and ionization with the vibrating probe. In addition, the use of a nanopipette simultaneously permitted a reduction in the sampling area and increased the ionization efficiency.^13)^

To further improve both the spatial resolution of the sampling and the sampling/ionization efficiency for mixtures of multiple chemical components inside tissues, it is necessary to understand the behavior of the nanoliter volumes of charged solvents provided by the capillary probe during sampling and ionization. However, the correlation between the formation and breakage of the liquid bridge and the subsequent ESI at the end of the probe is not yet completely understood.

In this study, we developed both an optical technique for measuring the displacement of the probe in real time and an electrical technique for controlling the distance between the probe and sample surface on the micrometer scale. These techniques were applied to measure the time course of the probe displacement and the ion signal of analyte molecules simultaneously during a single sampling and ionization process using a static probe. We also investigated the influence of the solvent (methanol/water, ethanol/water, and 2-propanol/water) and its flow rate on the formation and breakage of the liquid bridge and ESI.

## EXPERIMENTAL

### SPESI instrumentation

The device configuration used in this study was similar to that described in the previous report^8)^ except for the incorporation of both the electronic control system for adjusting the position of sample stage and the optical method for detecting probe displacement. The single event of sampling and ionization was controlled by varying the distance between the probe and sample surface. The capillary probe (FS360-20-10-N-20-C7, 10 μm i.d., 20 μm o.d., New Objective) was fixed at an angle of 45° relative to the sample stage. The sample was fixed on the XYZ stages (stepper motor stage: HPS60-20X, OSMS40-5ZF, Sigmakoki; piezo stage: APA120S, Cedrat Technologies), which were controlled using a custom-built program (LabVIEW, National Instruments). The time course of probe displacement and the mass spectrum were independently and simultaneously measured during a single sampling and ionization process. A syringe pump (Pump 11, Harvard Apparatus) was used to control the flow rate of solvents. A bias voltage of 3000 V was applied to the solvent through the syringe needle. A mass spectrometer (JMS-T100LP, JEOL) with the original MS inlet, in which the stainless steel tube (195 mm length, 2 mm opening diameter) was connected to orifice 1, was used. The voltage applied to the MS inlet was 80 V. The applied voltages to the ring lens, the orifice 1, and the orifice 2 were 20, 70 and 10 V, respectively. A homemade heater block (250°C) was attached to the stainless steel tude of MS inlet to desolvate the charged droplets generated by ESI. The probe position was fixed for all measurements using three solvents at the same flow rate.

### Optical method for measuring the probe displacement

To measure the probe displacement, the probe was irradiated with laser light (DMRS-20S-P2.6, Neoark Corp.) and the reflected light was measured using a quadrant photodiode (S5981, Hamamatsu Photonics). The voltage difference between the upper and lower halves of the photodiode was measured using a custom-built differential amplifier. To perform the measurement for a single sampling and ionization event, the distance between the probe and sample surface was controlled using the piezo stage. Initially, the sample surface was separated from the probe. The stage was moved toward the probe at a rate of 170 V/s. The conversion factor of piezo stage was 0.9 μm/V. The probe displacement was monitored during the movement of the sample stage. As the probe displacement signal reached a set value, at which the probe experienced a repulsive force, the stage was stopped and then it was retracted from the probe at the same speed as it had approached. Observations were performed for 10 s during this process. The XY stage was scanned at intervals of 100 or 200 μm to obtain measurements at different positions. After the measurements, the sampled spots were observed using an optical microscope (BX51, Olympus Corp.). The time course of probe displacement was analyzed using a custom-built program. A baseline was determined from the average value of the displacement signal 1 s prior to the probe touching the sample surface, at which time the probe had little interaction with the sample surface. Next, two time intervals were measured, namely, the extension time of the liquid bridge (*t*_1_) and the time from the breakage of the liquid bridge to the aquisition of the signal intensity of rhodamine B ion (*m*/*z* 443) by MS (*t*_2_). Data obtained from 30 different measurement points for each solvent, where the rhodamine B signal intensity exceeded 2000 arb. unit, were used for the analysis.

### Dye molecular film

A thin film of rhodamine B on a glass substrate was prepared as follows. A glass slide (S1112, Matsunami Corp.) was cut to dimensions of approximately 12 mm×12 mm. The glass substrates were successively ultrasonically cleaned (AU-80C, Aiwa Medical Industry Co.) for 15 min with ultrapure water (Direct-Q 3UV, Millipore) and acetone (00309-35, Nacalai Tesque Corp.), followed by UV ozone cleaning (UV253, Laser Techno Co., Ltd.) for 1 h. Finally, 150 μL of an ethanolic solution of rhodamine B (10 mM) was spin coated onto the cleaned glass at 4000 rpm for 30 s (MS-A100, Mikasa Corp.).

### Solvents

Three solvents were used for the analysis, namely, methanol (138-06473, FUJIFILM Wako Pure Chemical Industries, Ltd. Corp.), ethanol (057-00456, FUJIFILM Wako Pure Chemical Industries, Ltd. Corp.), and 2-propanol (29113-66, Nacalai Tesque Corp.), which were mixed with water to a final concentration of 60% (v/v). Formic acid (066-00461, FUJIFILM Wako Pure Chemical Industries, Ltd. Corp.) was added to each of the alcohol/water mixtures to a final concentration of 0.1% (v/v).

## RESULTS AND DISCUSSION

### Probe displacement and ion signal during sampling and ionization

**Figure 1:**
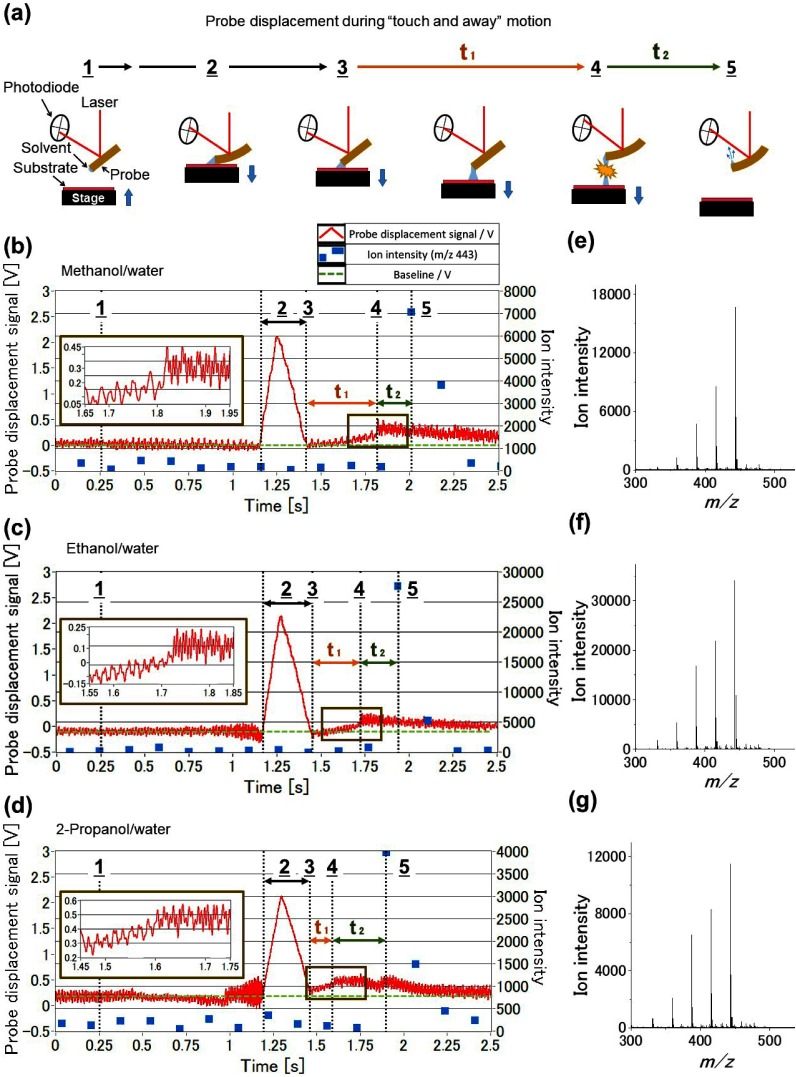
Fig. 1. (a) Schematic illustration of the measurement. (b)–(d) Time course of the probe displacement signal (solid red line), signal intensity for the rhodamine B ion (*m*/*z* 443; closed blue squares), and baseline (dashed green line) for the three solvents (methanol/water, ethanol/water, and 2-propanol/water). Magnified signals are shown in the inset, respectively. (e)–(g) Mass spectra obtained at the time 5 for (b)–(d).

Figure 1a shows a schematic diagram of the positional relationship between the probe and sample during a single sampling and ionization event. The probe displacement was observed using an optical detection technique, in which the laser light was focused on the side of the probe and the position of the laser light reflected from the probe surface was measured using a quadrant photodiode coupled to a differential amplifier. Prior to the measurement, the probe was distant from the sample (1) and a high voltage was applied to the solvent in the capillary to induce ESI. Next, the sample was moved toward the probe at a constant velocity. When the sample touched the probe, a liquid bridge was formed between the probe and sample surface. After the sample had made contact with the probe, the probe experienced a repulsive force and was pushed up by the sample. When the output signal from the optical method reached a set value corresponding to a particular repulsive force, the sample was retracted from the probe at the same speed as it had approached (2). During the separation of the substrate and probe tip (3), the liquid bridge first became extended (3–4) and then eventually broke (4). An enlarged view of the signal at break is shown in Fig. 1a. ESI then occurred from the probe tip (5).

The time courses of the MS ion signal and probe displacement signal were then compared. Figures 1b, c, and d show the results for the mixed solvents of methanol/water, ethanol/water, and 2-propanol/water, respectively. A thin film of rhodamine B on a glass substrate was used as the sample, and the flow rate of the solvent was 100 nL/min. In the plots, the solid lines, closed squares, and broken lines correspond to the probe displacement signal, the rhodamine B ion signal (*m*/*z* 443, [M−Cl]^+^), and the baseline indicating the neutral position of the probe prior to the sampling and ionization event. Figures 1e, f, and g show mass spectra at (5) for the mixed solvents of methanol/water, ethanol/water, and 2-propanol/water, respectively. The largest peaks (*m*/*z* 443) are [M–Cl]^+^. It is inferred that the four peaks appearing at intervals of *m*/*z* 28 are fragment ions of rhodamine B in which C_2_H_4_ groups are lost, respectively. A probe displacement signal lower or higher than the baseline indicates that the probe felt an attractive or repulsive force, respectively. The measurements for all three solvents indicated that the probe vibrated slightly when the probe and substrate were separated (1). The vibration frequency was approximately 60 Hz, which is lower than the resonant frequency of the probe (160 Hz). This vibration might be derived from the variation in solvent volume due to electrospray. A repulsive force was generated as the probe was pushed up by the sample (2). Since no vibration of the probe was observed during this contact, it is considered that the signal oscillation originated not from the electrical noise in the measurement system but from the vibration of the probe. Vibration of the probe was again observed during the stretching of the liquid bridge (3). Although the mechanism is unclear at present, the generation of a repulsive force between the probe and sample surface during the stretching of the liquid bridge was unexpected. This finding indicates the unique mechanical interactions occurring between the liquid bridge and probe. We observed the accumulated solvents at both the sample surface and probe tip, which are connected *via* the narrow liquid bridge by optical microscope. We assume the biased distribution of charged liquid resulted in an electrostatic repulsive force between the probe and sample surface. The vibration of the probe in this case might therefore be due to the opposing effects of the electrostatic repulsive force and surface tension. Finally, the probe vibrated at a higher frequency and larger amplitude immediately after the breakage of the liquid bridge (4), which was ascribed to the release of the pulling force between the probe and sample surface. Following the breakage of the liquid bridge, the repulsive force of the probe relaxed over time. This was ascribed to the consumption of the solution that had accumulated on the probe tip by ESI.

### Solvent effect on sampling and ionization processes

**Figure 2:**
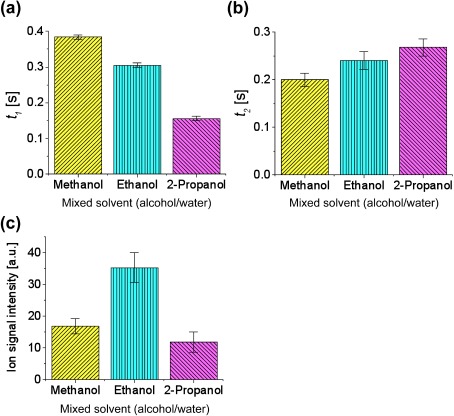
Fig. 2. Influence of solvent on the sampling and ionization process. (a) Variation of time needed to stretch the liquid bridge to breakage (*t*_1_). (b) Variation of time interval between the breakage of the liquid bridge and the detection of the rhodamine B ion signal (*t*_2_). (c) Variation of signal intensity for the rhodamine B ion (*m*/*z* 443).

To compare the results for the three different solvents, we defined the time to stretch the liquid bridge until breakage as *t*_1_, and the time from breakage of the liquid bridge to detection of the rhodamine B ion signal as *t*_2_. Figures 2a and b show the results for *t*_1_ and *t*_2_, respectively. For reference, Table 1 summarizes the viscosities^14)^ and surface tensions^15)^ of the 60% (v/v) alcohol/water mixed solvents and the vapor pressures^16)^ and dielectric constants^17)^ of the pure alcohols.

**Table 1:** Table 1. Physical properties of the solvents used and the results obtained in this study.

	Viscosity*^1^ [mPa·s]	Surface tension*^1^ [mN/m]	Dielectric constant*^2^	Vapor pressure*^2^ [Pa]	*t*_1_*^3^ [s]	*t*_2_*^3^ [s]	Ion intensity*^3^
Methanol	1.62	35.5	32.6	1.89×10^7^	0.384	0.200	1.68×10^4^
Ethanol	2.52	30.2	24.3	2.06×10^7^	0.305	0.241	3.53×10^4^
2-Propanol	3.70	25.3	18.3	2.07×10^7^	0.155	0.268	1.19×10^4^

*^1^: Mixed solvent. *^2^: Pure solvent. *^3^: Averaged values are obtained from the results of Fig. 2.

We observed that *t*_1_ decreased in the following order: methanol>ethanol>2-propanol. This order is positively correlated with the surface tension, indicating that the energy needed to break the liquid bridge is dominated by the surface energy of the liquid bridge. This is conceivable because the volume of the liquid bridge was estimated to be approximately 1–1.5 nL based on the contact time of the probe. On this scale, the ratio between the surface energy and internal energy of a liquid is larger than that for the bulk liquid.^18)^ Consequently, the greater the surface tension of the solvent, the longer the pulling time required to break the liquid bridge.

In contrast, *t*_2_ increased in the following order: methanol<ethanol<2-propanol. Following the breakage of the liquid bridge, the solution moved toward the MS inlet according to the electric field between the liquid captured at the probe tip and the MS inlet prior to ESI. During this process, the higher the viscosity of the solvent, the more time is needed to form the Taylor cone because the change of liquid shape is dependent on the viscosity.^19,20)^ The dielectric constant may also be responsible for this observation. As the dielectric constant of solvent increases, the Taylor cone stretches more rapidly with the electric field because the charge accumulation at the Taylor cone might be faster.

**Figure 3:**
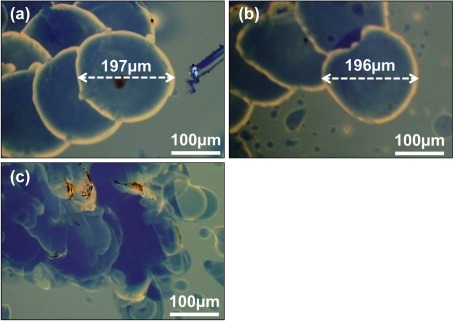
Fig. 3. Optical microscopy images of the sampled areas for the mixed solvents of (a) methanol/water, (b) ethanol/water, and (c) 2-propanol/water.

Figure 2c shows the signal intensity of the rhodamine B ion for the three mixed solvents. The signal intensity was highest for ethanol/water, followed by methanol/water and then 2-propanol/water. These results are considered in terms of both the sampling efficiency and ionization efficiency. The former is affected by the distribution of the liquid bridge on the sample surface to dissolve the sample components. To estimate the area of the liquid bridge, after the measurements the sampled areas were observed using an optical microscope. The sampled spots were found to be approximately the same size for methanol/water (Fig. 3a, 196 μm in diameter) and ethanol/water (Fig. 3b, 197 μm in diameter). These values are larger than the diameter of the probe aperture (10 μm), indicating that the solvent flowing from the probe had been distributed on the sample surface. The influence of the flow rate on the sampling area is discussed later.

In contrast, no distinct circular sampling spots were observed for 2-propanol/water (Fig. 3c). This may be attributable to a combination of the low surface tension and high vapor pressure of this solvent. The low contact angle of this solvent on the surface would result in the formation of a widely spread liquid bridge. Consequently, upon retracting the probe, the amount of rhodamine B remaining on the sample surface would be larger than that for the other two solvents. As a result, the amount of molecules subjected to ESI would be lower.

With respect to the ionization efficiency, this is known to be affected by the size of the charged droplets. It has been reported that the high charge density of small charged droplets is important for increasing the generation efficiency of gas-phase ions from the droplets.^21)^ For ESI, the size of a charged droplet can be estimated using the following equation.^22)^

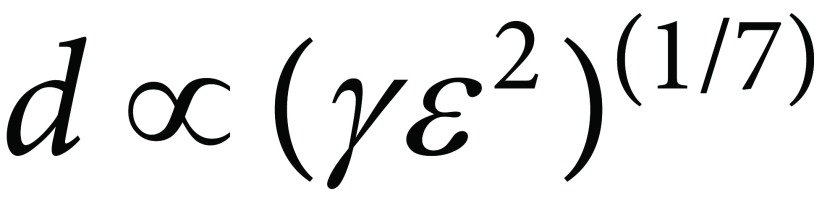
where γ is the surface tension, ε is the dielectric constant of the solvent, and *d* is the diameter of the droplet. Because γ and ε of ethanol are smaller than that of methanol (Table 1), the ethanol/water mixture would be expected to afford smaller droplets than would the methanol/water mixture. This would be expected to result in increased ionization efficiency.

### Flow rate dependence on sampling and ionization

**Figure 4:**
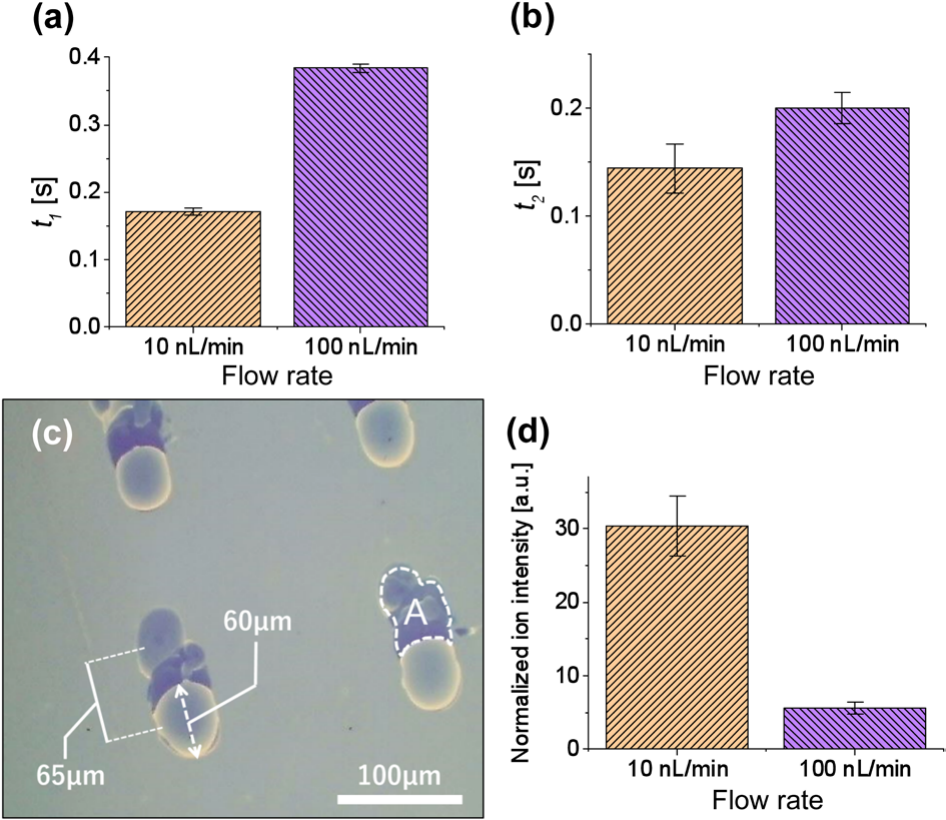
Fig. 4. Influence of flow rate on the sampling and ionization process for the mixed solvent of methanol/water. (a) Variation of time needed to stretch the liquid bridge to breakage (*t*_1_). (b) Variation of time interval between the breakage of the liquid bridge and the detection of the rhodamine B ion signal (*t*_2_). (c) Optical microscopy image of the sampled area at the flow rate of 10 nL/min. (d) Variation of signal intensity for the rhodamine B ion (*m*/*z* 443) normalized to the sampled area.

To evaluate the influence of the solvent flow rate on the sampling and ionization, measurements were conducted using different flow rates. Figure 4 shows the results for a low flow rate (10 nL/min) and a high flow rate (100 nL/min) of the methanol/water mixed solvent. Both *t*_1_ and *t*_2_ were shorter for the low flow rate than for the high flow rate (Figs. 4a and b). The sampled area was also smaller for the low flow rate (approximately 60 μm in diameter). Interestingly, overlapping sampling spots were observed in one direction (A in Fig. 4c). This is because the probe slid on the sample surface during the sampling process when the repulsive force was applied from the sample to the probe (2–3 in Fig. 1a). 

The distance between the sampled areas was 65 μm in the direction of the long axis, which is consistent with the displacement of the sample stage (*ca.* 50–60 μm) assuming a constant angle (45°) of the probe relative to the sample surface during sampling. To prevent the probe from sliding on the sample surface and realize a smaller sampling spot, a feedback system could be developed to reduce bending of the probe during the sampling process. Figure 4d shows a comparison of the intensity of the rhodamine B ion signal normalized by the sampled area. The signal intensity obtained using the flow rate of 10 nL/min was six times higher than that obtained using the flow rate of 100 nL/min. This result demonstrates the advantage of reducing the solvent volume to increase both the spatial resolution and the detection sensitivity during SPESI.

## CONCLUSION

In this study, we developed a visualizing system for probe motion during SPESI using a static capillary probe. We succeeded in simultaneously obtaining the time course of the probe displacement and the mass spectra during a single sampling and ionization process.

Experiments were performed for three different alcohol/water mixtures (methanol/water, ethanol/water, 2-propanol/water), which indicated that the time needed to stretch the liquid bridge until breakage was positively correlated with the surface tension of the solvent, and the time interval between the breakage and the detection of the ion signal was positively correlated with the solvent viscosity. It was also found that the ethanol/water mixture afforded the highest ion signal intensity of the three solvents. These results demonstrate that the physicochemical properties of the solvent influence both the liquid bridge formation and the size of the charged droplets during the sampling and ionization process. Moreover, measurements using the methanol/water mixture indicated the possibility of simultaneous improvement both of the sampling resolution and ionization efficiency due to the reduced volume of liquid bridge, which is controlled by the flow rate.

The measurement technique presented in this paper is important for understanding the dynamic properties of nanoliter charged liquid. The development of appropriate conditions for forming miniaturized liquid bridges is expected to open the door to achieving highly spatially resolved SPESI-MSI in the future.

## Notes

Mass Spectrom (Tokyo) 2018; 7(2): S0078
